# Influence of Simulated Radioactive Waste Resins on the Properties of Magnesium Silicate Hydrate Cement

**DOI:** 10.3390/ma18235385

**Published:** 2025-11-28

**Authors:** Enyu Sun, Huinan Gao, Min Li, Jie Yang, Yu Qiao, Tingting Zhang

**Affiliations:** 1 School of Applied Technology, University of Science and Technology Liaoning, Anshan 114051, China; lkdsuney@163.com; 2 Refractory Co., Ltd., Anshan I&S Group Co., Ltd., Anshan 114011, China; 3 The Building Technology Energy Conservation Service Center of Yuncheng, Yuncheng 044000, China; 13821623876@163.com; 4 Faculty of Infrastructure Engineering, Dalian University of Technology, Dalian 116023, China; qiaoyu@cbmi.com.cn; 5 College of Civil Engineering and Architecture, Zhejiang University, Hangzhou 310058, China; 6 CBMI Construction Co., Ltd., Beijing 100176, China

**Keywords:** magnesium silicate hydrate cement, ion exchange resins, immobilization, leaching, durability

## Abstract

Ion exchange resins are commonly utilized for treating liquid radioactive waste within nuclear power plants; however, the disposal of these waste resins presents a new challenge. In this study, magnesium silicate hydrate cement (MSHC) was used to immobilize the waste resin, and the immobilization effectiveness of the MSHC-solidified body were assessed by mechanical properties, durability, and leaching performance. Hydration heat, X-ray diffraction (XRD), thermogravimetric analysis (TGA), scanning electronic microscopy (SEM), and mercury intrusion porosimetry (MIP) were used to study the hydration process of the MSHC-solidified body containing Cs^+^, Sr^2+^, and Cs^+^/Sr^2+^ waste resins. The results demonstrated that the presence of waste resins slightly delayed the hydration reaction process of MSHC and reduced the polymerization degree of the M-S-H gel, and the composition of the hydration products were not changed. The immobilization mechanism for radionuclide ions in resin included both mechanical encapsulation and surface adsorption, and the leaching of Cs^+^ and Sr^2+^ from MSHC-solidified body followed the FRDIM. When the content of the waste resin was 25%, the MSHC-solidified body exhibited satisfactory compressive strength, freeze-thaw resistance, soaking resistance, and impact resistance. These results strongly indicated that MSHC possessed the ability to effectively immobilize ion exchange resins.

## 1. Introduction

With the rapid increase in energy demands and the problem of climate change, nuclear power is widely used all over the world, and has become the second largest source of low-carbon power generation [1]. However, nuclear power production generates a significant amount of radioactive waste [2]. Ion exchange resins find widespread application within nuclear power plants, serving for coolant purification and the treatment of liquid radioactive waste [3,4]. Once radionuclides are absorbed to their saturation point, the resulting waste resins become medium-level radioactive waste, requiring appropriate management and disposal [5]. Improper handling of this radioactive waste can pose significant risks to humans and other organisms [6,7].

Immobilization is a widely adopted approach for radioactive waste disposal. Therefore, numerous countries such as Japan, Korea and China have implemented this method to manage radioactive resins [2,8]. The immobilization techniques for radioactive waste resins primarily involve bituminization, vitrification, and cementation [5,9]. The saponification reaction of asphalt is used to treat waste resins, resulting in a solidified body of dried and dehydrated waste resins encapsulated by molten asphalt. This solidified waste resin has a low radionuclide leaching rate and a high resin package capacity. However, due to the flammability and the poor binding capacity of asphalt and the resin, the treatment process is danger prone, and blockage of radiation from the treated waste is poor [10,11]. Inert glass has good chemical resistance to ionic leaching and does not need additional protection or adaptive activity [12], and it can be directly disposed of in landfills [13]. However, the limited ion adsorption and suboptimal properties of waste treated with glass have hindered the development of this process [14]. In summary, cement immobilization has become the most promising method for immobilizing waste.

Cement has been widely used as the primary material for immobilizing waste resins due to its favorable physical and chemical properties, ease of use, and affordability [4,15,16]. Portland cement, a commonly used type of cement worldwide, has relatively high porosity and brittleness, necessitating the use of various auxiliary cementing materials or additives to enhance its immobilization effectiveness [4,17]. For instance, the presence of zeolite substantially can increase the retention rate of Cs^+^ [18]. Li et al. [19] utilized cement as the base material for immobilizing waste resins and added zeolite (20% of the total mixture) to create a high-performance matrix with high compressive strength, high resin loading, and a low leaching rate. Matsuda et al. [20] added steel fiber in the cement matrix (10% of the total mixture) and the resin content was increased from 20% to 40% of the mixture, which led to improved immobilization efficiency. Pan et al. [21] utilized Taguchi analysis to optimize the process of resin immobilization, revealing that the most favorable immobilization effect was achieved by incorporating 24% furnace slag and 24% fly ash into the cement. However, traditional cement, which has high consumption and carbon emissions, needs to be replaced by new cementitious materials [22].

Magnesium silicate hydrate cement (MSHC), a type of magnesium-based cement [23], holds great potential for the development of a low-energy, green, and low-carbon cement. Its low alkalinity (9.5–10.5) makes it a potential packaging material because of the lowest solubility of most heavy metals at the pH range of 9–12 [24,25]. Furthermore, the porous structure of MSHC grants it exceptional absorption capabilities [25]. Additionally, the rate of ion leaching from MSHC-solidified bodies is lower compared to OPC-solidified bodies [26]. Fouad and Bishay [27] discovered that synthetic magnesium silicate can extract and treat 98% of radioactive uranium (UO_2_(NO_3_)_2_·6H_2_O) in acidic solutions. Wang et al. [28] successfully recovered polluted sediment using a magnesium-silicon system and demonstrated satisfactory results after fixing the toxic elements. Specifically, the mechanical properties of the solidified pollutants were found to be satisfactory. Walling et al. [29] showed that the Mg(OH)_2_-SiO_2_-H_2_O system, using M-S-H gel as a hydration product, can immobilize radioactive sludge from a Magnox nuclear power plant. Their work provided a methodology for testing the immobilization of simulated nuclear waste using MSHC.

Although there have been some studies on using MSHC to immobilize the toxic and radioactive substances, the application of MSHC to handle used ion exchange resins containing radioactive substances has not garnered attention from scholars. Thus, in this study, we evaluated the immobilization effectiveness of MSHC on simulated radioactive waste resins, considering various parameters including compressive strength, durability and leaching performance. This research delves into the novel investigation of MSHC as an immobilization agent for waste resins containing Cs^+^ and Sr^2+^, presenting a new disposal solution for decommissioned radioactive waste resins. The findings hold significant theoretical guidance for future practical applications.

## 2. Materials and Methods

### 2.1. Materials and Sample Preparation

The MSHC was prepared using light-burned magnesium oxide (MgO, Hebei Qinghe Xinhu Metal Materials Company Limited, Xingtai, China) with 85% activity, silica fume (SF, 940U, Elkem, Oslo, Norway) with SiO_2_ content >97%, sodium hexametaphosphate (SHMP, China National Pharmaceutical Group Corporation, Beijing, China), and quartz sand. The chemical composition of MgO and SF was shown in Table 1.

The preparation process of simulated radioactive waste resins from non-radioactive ion exchange resins was as follows: Firstly, CsCl and Sr(NO_3_)_2_ were added to a saturated boric acid solution at a concentration of 0.025 mol/L to replace the radionuclides, Cs and Sr, respectively. In this process, boric acid was used to simulate the boric acid ions adsorbed by the ion exchange resin in a nuclear power plant. Then, the original resin was mixed with the solution and soaked in a mass ratio of 3:4 for 7 days. After that, the waste resin was drained through a 200-mesh sieve until no water dripped out.

When the molar ratio of MgO to SiO_2_ was 1, and the SHMP dosage was 2% of the mass of MgO and SF, MSHC was reported to exhibit good mechanical properties [30,31]. To prepare the MSHC, the corresponding raw materials were weighed to achieve a water-cement ratio of 0.5, a cement-sand ratio of 1:3, and a 25% waste resin content. The slurry was stirred and poured into molds (Φ50 mm × 50 mm cylinders), cured for 1 day, and then demolded. The cured slurry was additionally subjected to curing for the required ages at a temperature of 20 ± 2 °C and a relative humidity of ≥ 95%.

### 2.2. Experimental Methods

According to GB 14569.1-2011 [32], the compressive strength, impact resistance, freeze-thaw resistance, and soaking resistance of the MSHC-solidified body (the cured slurry) after 28 days were studied. The compressive strength was measured using a microcomputer testing machine (WHY-300/10, Shanghai Hualong Testing Machine Co., Ltd., Shanghai, China). There were six samples for each material, and the average was taken as the compressive strength result. For the impact test, the solidified body was dropped from a height of 9 m onto a concrete floor, and the appearance and integrity of the solidified body were recorded. The freeze-thaw resistance test involved placing the solidified body in a sealed plastic bag and freezing it at −20 °C ~ −15 °C for 3 h. Then, the bagged solidified body was placed in a water tank at 15 °C ~ 20 °C for 4 h. After five of these freeze-thaw cycles, the appearance and integrity of the solidified body were observed and its compressive strength were tested. The soaking resistance test involved soaking the solidified body in deionized water for 90 days, observing any cracks in the appearance, and comparing the compressive strength after soaking to the compressive strength before soaking.

In the leaching test, the test block was polished on its upper and lower surfaces and then suspended in a polyethylene bottle using a nylon rope. A volume of 1.2 L of deionized water or artificial seawater was added as a leaching agent. The test was conducted at a temperature of 25 ± 2 °C and 40 ± 2 °C. Five-milliliter samples of the leaching solution were collected on days 1, 3, 7, 10, 14, 21, 28, 35 and 42, and then the solution was replaced with fresh solution after each collection. The concentration of Cs^+^ and Sr^2+^ in each leaching period sample was measured using a plasma emission spectrometer, and the leaching rate and cumulative leaching fraction of Cs^+^ and Sr^2+^ were calculated following the method described by Lu et al. [33]:

(1) Rn=anA0SV∆tn

(2) Pt=∑anA0SV

where *R*_n_ represents the leaching rate of Cs^+^ and Sr^2+^ during the nth leaching cycle (cm/d), *a*_n_ stands for the leaching mass of Cs^+^ and Sr^2+^ during the nth leaching cycle (g), *A*_0_ denotes the initial mass of Cs^+^ and Sr^2+^ (g), *S* signifies the geometric surface area of the sample exposed to the leaching agent (cm^2^), *V* represents the sample volume (cm^3^), *(∆t)*_n_ corresponds to the duration of the nth leaching cycle (d), *P*_t_ represents the cumulative leaching fraction of Cs^+^ and Sr^2+^ at leaching time *t* (cm), and *t* is the cumulative leaching days (d).

The cement hydration microcalorimeter (Tam Air III, TA Instruments, New Castle, DE, USA) was used to test the heat flow rate and cumulative heat flow from the hydration reaction that occurred during the mixing of raw materials. X-ray diffraction (XRD, D8 advance, Bruker, Karlrushe, Germany) was used for qualitative analysis of the solidified product’s phase composition. Thermogravimetric analysis (TG, TGA-DSC 1, Mettler-Toledo, Greifensee, Switzerland) was used to analyze the contents of Mg(OH)_2_ and M-S-H gel in the solidified body. Scanning electron microscopy (Nova Nano SEM-50, HITACHI, Tokyo, Japan) was used to analyze the micromorphology of the solidified body. Mercury intrusion porosimetry (MIP, AutoPore IV9500, Micromeritics, Noklossville, GA, USA) was used to analyze the pore structure of the sample.

## 3. Results

### 3.1. Mechanical Properties

#### 3.1.1. Compressive Strength

**Figure 1:**
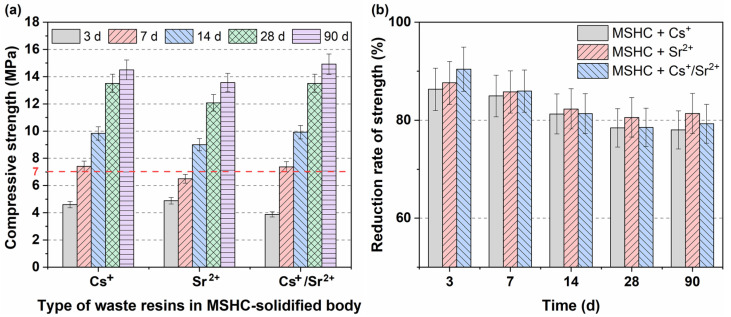
(**a**) Compressive strength, and (**b**) reduction rate of compressive strength of different types of waste resins at different ages.

Figure 1a shows the compressive strength of the MSHC-solidified body containing Cs^+^, Sr^2+^, and Cs^+^/Sr^2+^ at different ages. After immobilizing the waste resin, the compressive strength of the solidified body was notably lower than that of MSHC. The 28-day compressive strength of all solidified bodies exceeded 7 MPa. Notably, the solidified body containing Cs^+^ and Cs^+^/Sr^2+^ achieved this strength within just 7 days. During the first 3 days, the compressive strength of the solidified body containing Sr^2+^ increased fast. When it was cured for 7 days, its compressive strength was exceeded by the solidified body containing Cs^+^ and Cs^+^/Sr^2+^. After 7 days, the compressive strength ranking remained as follows: Cs^+^/Sr^2+^ > Cs^+^ > Sr^2+^. The compressive strength of the solidified body demonstrated an increase over time. At 90 days, the solidified body containing Cs^+^, Sr^2+^, and Cs^+^/Sr^2+^ reached 14.43 MPa, 13.49 MPa, and 14.88 MPa, respectively.

Figure 1b illustrates the reduction rate of compressive strength for MSHC-solidified bodies containing waste resins at different ages. The compressive strength of a MSHC-solidified body devoid of waste resins served as the blank control for comparison. With the progression of curing time, the reduction rate of compressive strength showed a slight decrease. In the same curing age, the influence of different radionuclide ions in the waste resin on the reduction rate of compressive strength was not significant. At 3 days, the compressive strength reduction rates of waste resins containing Cs^+^, Sr^2+^, and Cs^+^/Sr^2+^ were 86.31%, 87.85%, and 90.26%, respectively, while at 90 days, the reduction rates were 78.19%, 81.43%, and 79.36%, respectively.

#### 3.1.2. Impact Resistance

**Figure 2:**
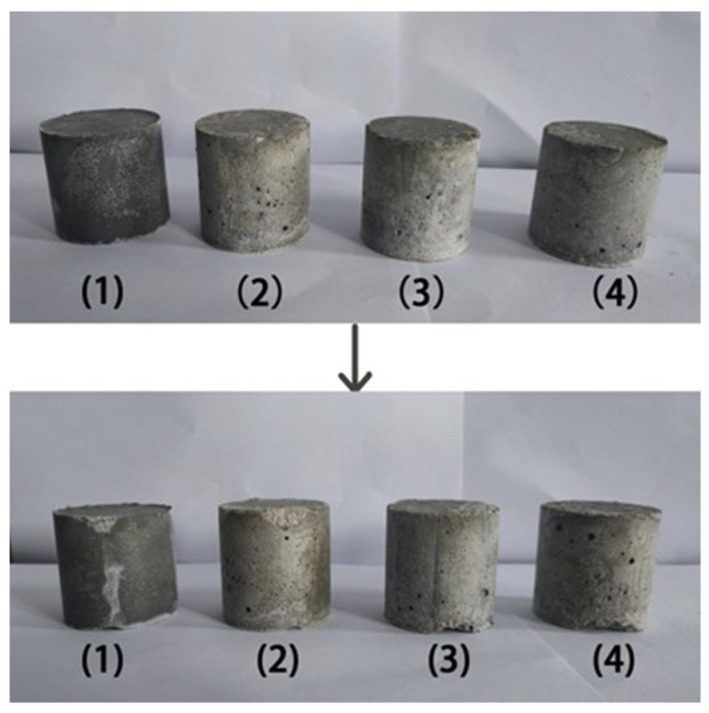
Appearance and morphology of the MSHC-solidified body containing waste resins before and after the impact test: (1) MSHC, (2) MSHC + Cs^+^, (3) MSHC + Sr^2+^, and (4) MSHC + Cs^+^/Sr^2+^.

The anti-impact test was conducted to simulate a scenario where the solidified body fell from a vehicle traveling at 50 km/h during transportation. Figure 2 demonstrates the appearance and morphology of the MSHC-solidified body containing Cs^+^, Sr^2+^, and Cs^+^/Sr^2+^ waste resins before and after the impact test. The photographs revealed that the solidified bodies treated with MSHC retained their appearance and integrity, with no significant cracks observed. Only small fragments came off from the upper and lower edges of the cylinders due to the impact. In addition, the solidified body containing waste resins exhibited fewer defects than MSHC after being subjected to impact. Among the solidified bodies, the one containing Sr^2+^ suffered the most severe damage.

### 3.2. Durability

#### 3.2.1. Freeze-Thaw Resistance

**Figure 3:**
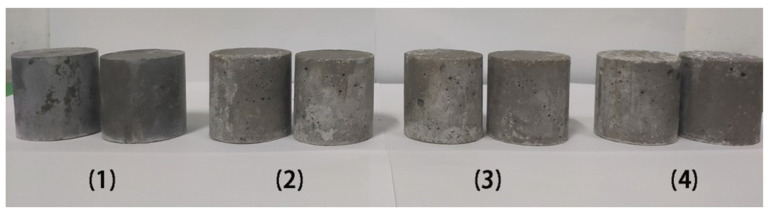
Appearance and morphology of the MSHC-solidified body containing waste resins after the freeze-thaw test: (1) MSHC, (2) MSHC + Cs^+^, (3) MSHC + Sr^2+^, and (4) MSHC + Cs^+^/Sr^2+^.

The appearance and morphology of the MSHC-solidified body containing Cs^+^, Sr^2+^, and Cs^+^/Sr^2+^ waste resins after the freeze-thaw test are shown in Figure 3. The solidified body maintained good integrity after five freeze-thaw cycles, with no apparent cracks formed. Table 2 presents the mass loss rate and the compressive strength loss rate of the solidified body. The results indicated that the incorporation of waste resins enhanced the mass loss rate, while also escalating the reduction in compressive strength. The compressive strength loss rate of the solidified body containing Sr^2+^ waste resins was the lowest, similar to MSHC, while the solidified bodies containing Cs^+^ and Cs^+^/Sr^2+^ waste resins exhibited a higher strength loss rate. The ranking of mass loss rate differed slightly from that of compressive strength loss rate, with the highest value found in the solidified body containing Cs^+^/Sr^2+^, while the values of Cs^+^ and Sr^2+^ were relatively close to each other. In general, the mass loss rates remained below the 5% limit, and the compressive strength loss rates remained below the 25% limit.

#### 3.2.2. Soaking Resistance

**Figure 4:**
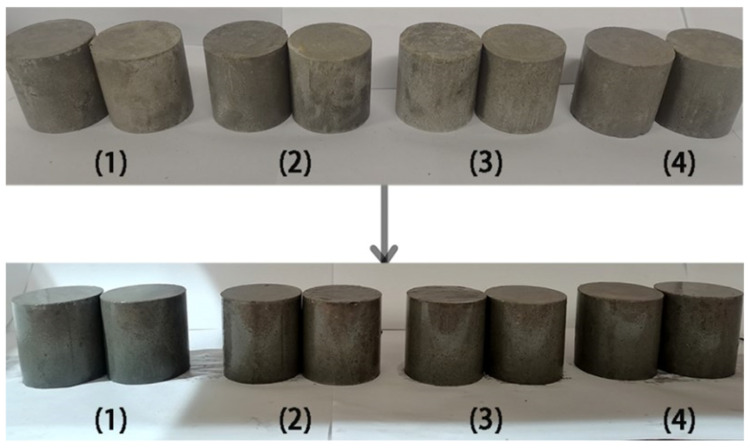
Appearance and morphology of the MSHC-solidified body containing waste resins before and after the soaking test: (1) MSHC, (2) MSHC + Cs^+^, (3) MSHC + Sr^2+^, and (4) MSHC + Cs^+^/Sr^2+^.

The appearance and morphology of the MSHC-solidified body containing Cs^+^, Sr^2+^, and Cs^+^/Sr^2+^ waste resins before and after the soaking test are demonstrated in Figure 4. It showed that the appearance of the solidified body remained intact without any visible signs of cracking or breaking after the soaking test. The compressive strength ratio of the solidified body before and after soaking are presented in Table 3. It indicated that the compressive strength of the solidified body after soaking increased. Particularly, for the solidified body containing Cs^+^/Sr^2+^ waste resins, the compressive strength after soaking was 1.25 times higher than before soaking, which exceeded the growth rate observed in MSHC. After soaking, the strength of the solidified body containing Cs^+^, Sr^2+^, and Cs^+^/Sr^2+^ waste resins remained above 7 MPa.

### 3.3. Leaching Performance

**Figure 5:**
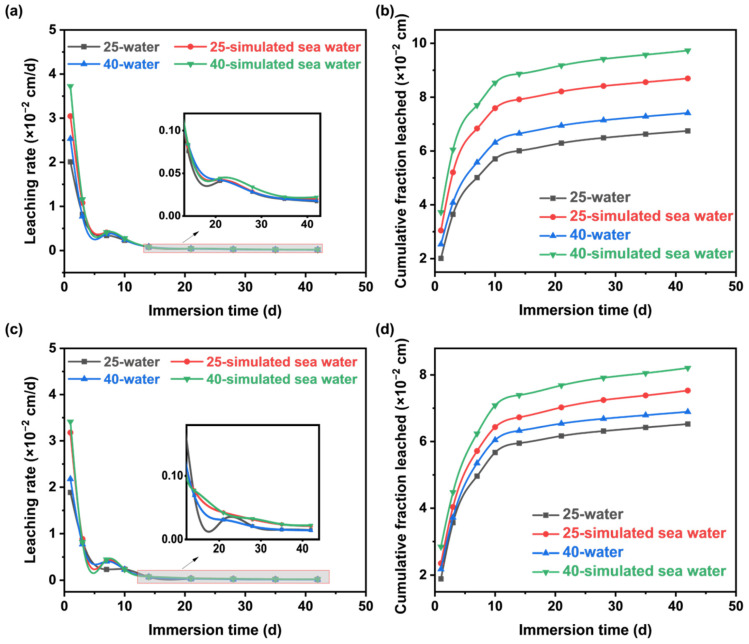
Leaching rate and cumulative leaching fraction of the MSHC-solidified body containing waste resins: (**a**) Cs^+^ leaching rate, (**b**) Cs^+^ cumulative leaching fraction, (**c**) Sr^2+^ leaching rate, and (**d**) Sr^2+^ cumulative leaching fraction.

The leaching rate and cumulative leaching fraction of Cs^+^ and Sr^2+^ in the MSHC-solidified body containing Cs^+^ and Sr^2+^ waste resins are illustrated in Figure 5. All solidified bodies remained intact without any changes in volume or shape during the leaching process. The leaching rates of Cs^+^ and Sr^2+^ under various leaching conditions can be classified into three stages based on leaching time: during the initial 1–7 days, the ion leaching rate experienced a rapid decrease, with significant differences observed among different leaching conditions; from 7 to 28 days, the decrease in ion leaching rate slowed down; and after 28 days of leaching, the leaching rate gradually stabilized, with relatively minor differences in ion leaching rate among different leaching conditions. As the leaching time increased, the cumulative leaching fraction of Cs^+^ and Sr^2+^ exhibited a rapid increase followed by a gradual stabilization. The most significant differences in ion leaching rates were observed under different leaching conditions during the first stage.

At 1–3 days of leaching, the leaching rates of Cs^+^ and Sr^2+^ in the MSHC solidified body were lower at 25 °C than at 40 °C. Between 3–7 days of leaching, the influence of temperature on the leaching rate of Sr^2+^ showed slight fluctuations compared to the previous 3 days, while the effect on Cs^+^ remained unchanged. However, after 7 days of leaching, the temperature had almost no effect on the leaching rate of both elements. Subsequently, after 42 days of leaching, the leaching rates of Cs^+^ and Sr^2+^ in the MSHC-solidified body at different temperatures were found to be lower than the limit requirements specified in the standard, which were 4 × 10^−3^ cm/d and 1 × 10^−3^ cm/d, respectively. The cumulative leaching fractions of Cs^+^ and Sr^2+^ rose with the immersion time and temperature increased. After 42 days of leaching, the solidified body exhibited the highest cumulative leaching fraction in the 40 °C environment. However, even in this case, the cumulative leaching fractions were still below the limit requirements of 0.26 cm (Cs^+^) and 0.17 cm (Sr^2+^) [32].

Under the condition of 25 °C and leaching for 1–21 days, the leaching rates of Cs^+^ and Sr^2+^ in a seawater environment were higher. After 21 days of leaching, the influence of the leaching agent on the leaching rate of Sr^2+^ remained unchanged, and the leaching rate of Cs^+^ was no longer affected by the leaching agent. At 40 °C, only during the 14 to 21 days of leaching, the leaching rate of Cs^+^ in deionized water exceeded that in seawater. At other times, the leaching rates of Cs^+^ and Sr^2+^ in deionized water were lower than those in simulated seawater. After 42 days of leaching, the leaching rates of Cs^+^ and Sr^2+^ in the solidified body under different leaching agent conditions were all lower than the limit requirements, at 4 × 10^−3^ cm/d and 1 × 10^−3^ cm/d, respectively. When leaching for 42 days in a seawater environment, the cumulative leaching fraction of Cs^+^ and Sr^2+^ in the solidified body reached their maximum values. However, they were still below the limit requirements specified in the standard of 0.26 cm and 0.17 cm, respectively [32].

### 3.4. Microstructural Analyses

#### 3.4.1. Hydration Heat

**Figure 6:**
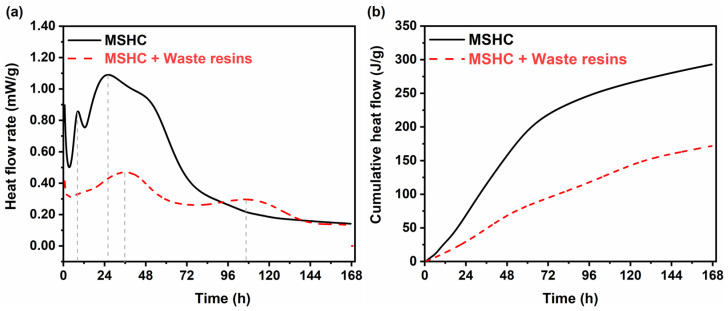
(**a**) Heat flow rate, and (**b**) cumulative heat flow of the MSHC-solidified body containing waste resins.

The heat flow rate and cumulative heat flow during the hydration reaction are indicators that can be used to analyze the progress of the reaction. The heat flow rate and cumulative heat flow curves over 7 days for the MSHC blank control group and the experimental groups containing 25% waste resins are illustrated in Figure 6. The first exothermic peak of the MSHC was primarily due to the wet dissolution of the raw material, and the exothermic rate was associated with the specific surface area of the raw material particles. The hydration reaction lasted for approximately 26 h, and a second peak appeared in the formation of hydration products Mg(OH)_2_ and M-S-H gel. After immobilizing the simulated waste resins, the appearance time of the two exothermic peaks of the MSHC was significantly delayed, both extended by approximately threefold. This suggested that the hydration reaction of the MSHC was delayed after the waste resin was immobilized. After immobilizing the simulated waste resins, the cumulative heat flow during the hydration reaction was reduced, and the heat release process became gentler.

#### 3.4.2. XRD

**Figure 7:**
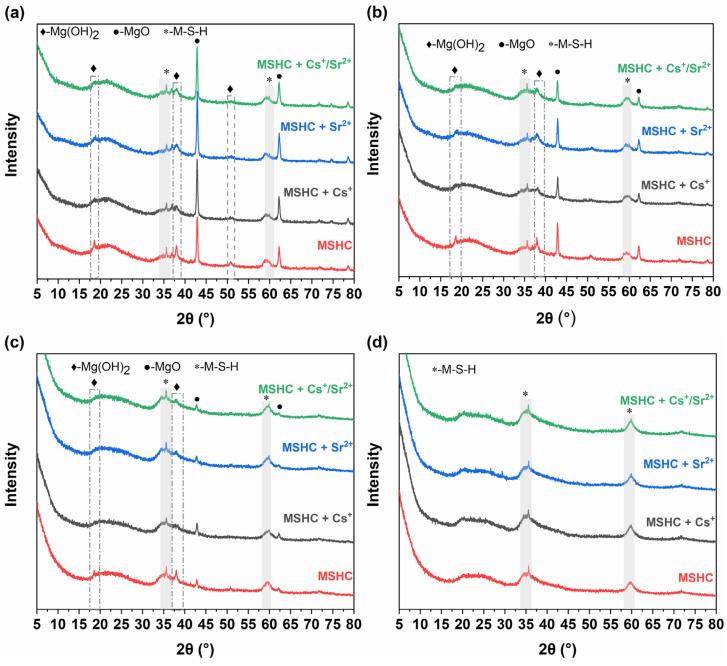
XRD patterns of the MSHC-solidified body containing waste resins at different ages: (**a**) 3 days, (**b**) 7 days, (**c**) 28 days, and (**d**) 90 days.

The XRD patterns of the MSHC-solidified bodies containing Cs^+^, Sr^2+^, and Cs^+^/Sr^2+^ waste resins at different ages are shown in Figure 7. After curing for 3 days, the MSHC mainly consisted of unreacted MgO, while a small amount of Mg(OH)_2_ and M-S-H gel were also formed. With the addition of waste resins, the hydration reaction was delayed, resulting in a weak Mg(OH)_2_ diffraction peak. As the reaction progressed for 7 days, MgO continued to dissolve and react, leading to the formation of Mg(OH)_2_, and the diffraction peak of unreacted MgO gradually weakened. With the reaction of magnesium ions and silicate ions, Mg(OH)_2_ was further converted into M-S-H gel, and the diffraction peak of Mg(OH)_2_ in the MSHC-solidified body containing waste resins remained weak, indicating a slow hydration reaction rate. After curing for 28 days, MgO in the MSHC was mostly dissolved, and the main product was M-S-H gel, with a small amount of Mg(OH)_2_. At 90 days, the main product was M-S-H gel, and the incorporation of waste resins did not produce a new phase in the MSHC. The hydration products were still M-S-H gel and Mg(OH)_2_, but the relative content may have changed.

#### 3.4.3. TG

**Figure 8:**
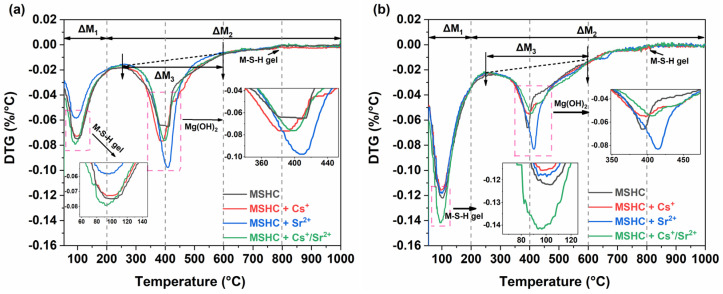
DTG diagram of the MSHC-solidified body containing waste resins at different ages: (**a**) 7 days, and (**b**) 28 days.

The content of Mg(OH)_2_ in the MSHC can be quantitatively analyzed by TG, while the M-S-H gel can be characterized by the content of physically adsorbed water and chemically bound water in the derivative thermogravimetric (DTG) curve [34]. Figure 8 illustrates the DTG curve of the MSHC-solidified body containing Cs^+^, Sr^2+^, and Cs^+^/Sr^2+^ waste resins at 7 days and 28 days.

The content of Mg(OH)_2_ and M-S-H gel was quantitatively studied using a DTG curve. The calculation formula was as follows:

(3) $$C\left[{Mg\left(OH\right)}_{2}\right]=∆M_{3}\times \frac{M_{\left[{Mg\left(OH\right)}_{2}\right]}}{M_{\left[H_{2}O\right]}}$$

(4) $$C{\left[PAW\right]}_{M-S-Hgel}=∆M_{1}$$

(5) $$C{\left[CBW\right]}_{M-S-Hgel}=∆M_{2}-∆M_{3}$$

(6) $$C\left[M-S-H\;gel\right]=∆M_{1}+∆M_{2}-∆M_{3}$$

where *C*[*Mg(OH)*_2_] and *C*[*M-S-H gel*] are the content of Mg(OH)_2_ and M-S-H gel in the solidified body (%), *∆M*_3_ is the loss of hydroxy water in Mg(OH)_2_ (%), *M*_[Mg(OH)2]_ and *M* _[H2O]_ are the molar mass of Mg(OH)_2_ and H_2_O (g/mol), *C*[*PAW*]_M-S-H gel_ and *∆M*_1_ are the loss of physically adsorbed water (%), *C*[*CBW*]_M-S-H gel_ is the loss of M-S-H gel chemically bound water (%), and *∆M*_2_ is the loss of hydroxy water in Mg(OH)_2_ and M-S-H gel chemically bound water (%). Utilizing the provided formula, the contents of Mg(OH)_2_ and M-S-H gel in the MSHC-solidified body containing Cs^+^, Sr^2+^, and Cs^+^/Sr^2+^ waste resins can be calculated when the curing time was 7 days and 28 days, respectively, as shown in Table 4.

With the progression of curing time from 7 to 28 days, the mass loss of physically adsorbed water of M-S-H gel increased, while the mass loss caused by the dihydroxylation of Mg(OH)_2_ decreased, indicating a gradual transformation of Mg(OH)_2_ into M-S-H gel over the curing period. Additionally, the content of Mg(OH)_2_ in the solidified body containing waste resins surpassed that in the control group, accompanied by a decrease in the content of M-S-H gel. Combined with the hydration heat curve and XRD text results, Mg(OH)_2_ in the MSHC began to transform into M-S-H gel, while the MSHC-solidified body containing waste resins mainly consisted of Mg(OH)_2_ due to the delayed hydration reaction. Therefore, compared with the blank group, the amount of Mg(OH)_2_ was higher, while the content of M-S-H gel was lower. After curing for 28 days, the main reaction product in the blank group was M-S-H gel, constituting approximately 1.74 times the amount of Mg(OH)_2_ present. Among the MSHC-solidified body containing waste resins, the proportion of M-S-H gel, which served as a strength-supporting component, decreased, measuring only 1.44, 1.13, and 1.37 times the amount of Mg(OH)_2_.

#### 3.4.4. SEM

**Figure 9:**
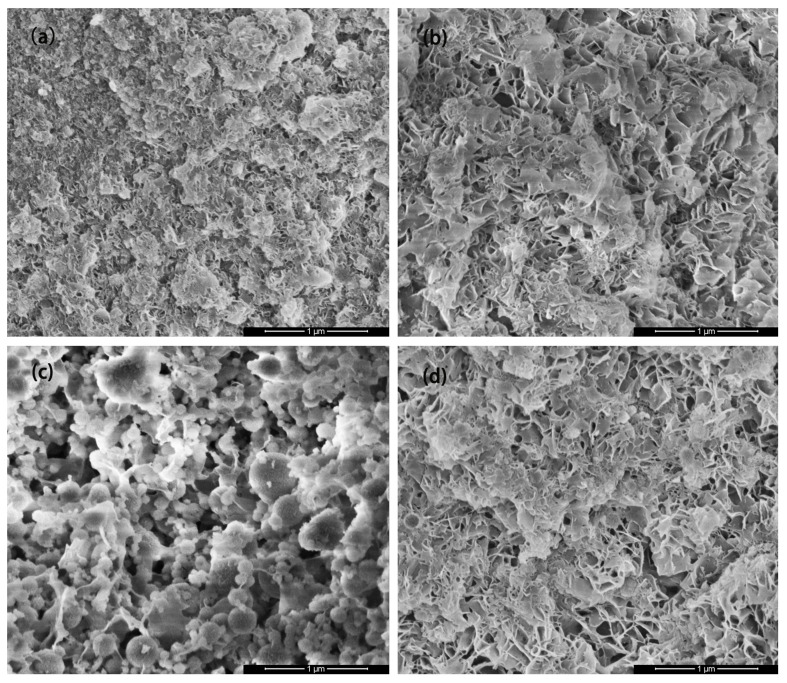
SEM images of the MSHC-solidified body containing waste resins at 3 days: (**a**) MSHC, (**b**) MSHC + Cs^+^, (**c**) MSHC + Sr^2+^, and (**d**) MSHC + Cs^+^/Sr^2+^.

The microstructure of the MSHC-solidified body containing Cs^+^, Sr^2+^, and Cs^+^/Sr^2+^ waste resins at 3 days are demonstrated in Figure 9. At 3 days of curing, smaller-sized flake-like Mg(OH)_2_ was the predominant component of the MSHC and was partially covered by generated M-S-H gel. However, upon the inclusion of waste resins containing Cs^+^, and Cs^+^/Sr^2+^, the size of flake-like Mg(OH)_2_ underwent a notable increase, while the content of M-S-H gel exhibited a reduction. This indicated that the solidified body relied solely on the overlapping of sheets and had more pores. Furthermore, for the MSHC containing waste resins with Sr^2+^, there were granular particles of MgO and SiO_2_, suggesting that the hydration reaction of the MSHC was delayed by the addition of waste resins, particularly those containing Sr^2+^.

**Figure 10:**
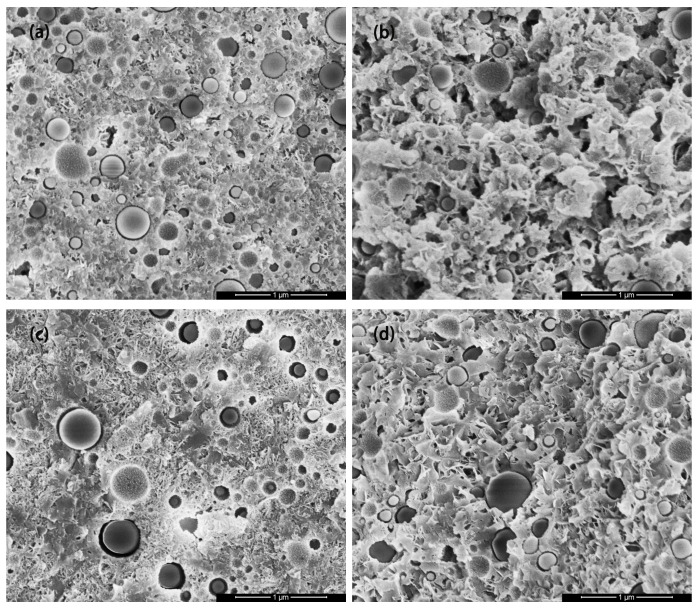
SEM images of the MSHC-solidified body containing waste resins at 28 days: (**a**) MSHC, (**b**) MSHC + Cs^+^, (**c**) MSHC + Sr^2+^, and (**d**) MSHC + Cs^+^/Sr^2+^.

The microstructure of the MSHC-solidified body containing Cs^+^, Sr^2+^, and Cs^+^/Sr^2+^ waste resins at 28 days are illustrated in Figure 10. With the ongoing hydration reaction, Mg(OH)_2_ gradually transformed into M-S-H gel. After 28 days of curing, the main product M-S-H gel wrapped around SF particles, and the flocculent gels overlapped and reunited to form a dense microstructure. However, after adding waste resins, the hydration products were primarily composed of M-S-H gel, but flake-like Mg(OH)_2_ still persists, and the degree of polymerization may have changed. Additionally, the degree of interweaving between the products was lower, resulting in a looser microstructure compared to the MSHC. No new crystal products were found in the microstructure at each age.

#### 3.4.5. MIP

The results of pore structure analysis for the MSHC-solidified body containing Cs^+^, Sr^2+^, and Cs^+^/Sr^2+^ waste resins are showed in Table 5. The data showed that the median pore diameter in the solidified body of the original MSHC was 7.2 nm, with a porosity of 15.28%, indicating that the MSHC mainly consisted of gel pores. With the addition of waste resins, the porosity of the solidified body increased by 1.48 times, 1.76 times, and 1.30 times, respectively, compared to the MSHC.

**Figure 11:**
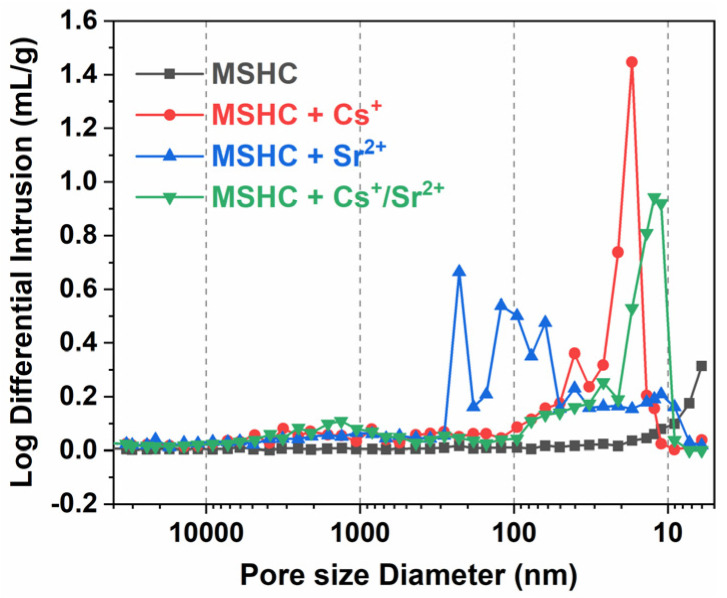
Pore size distribution diagram of the MSHC-solidified body containing Cs^+^, Sr^2+^, and Cs^+^/Sr^2+^ waste resins at 28 days.

Figure 11 shows the pore size distribution diagram of the MSHC-solidified body containing Cs^+^, Sr^2+^, and Cs^+^/Sr^2+^ waste resins at 28 days. In the solidified body containing waste resins with Cs^+^ and Cs^+^/Sr^2+^, the pores with a diameter of 10-50 nm increased noticeably, while those with a diameter of 50–300 nm increased more in the solidified body containing Sr^2+^. The increase in harmful pores significantly decreased the compressive strength of the solidified body. Among the solidified bodies, the one containing Sr^2+^ exhibited the lowest strength, with the most substantial reduction rate at 28 days.

## 4. Discussion

### 4.1. Impact of Waste Resins on MSHC

#### 4.1.1. Influence of Incorporating Waste Resins on the Mechanical and Durability Performance of the MSHC-Solidified Body

In this study, MSHC was used to immobilize ion exchange resin for the first time. MSHC is chosen due to its simpler production process, lower carbon dioxide emissions and superior immobilization effectiveness compared to ordinary cement [26,35,36]. The strength of MSHC-solidified body containing waste resins exceeds 7 MPa after 28 days, this value can ensure the solidity of the solidified body, thereby reducing the potential threat. The hydration heat release of MSHC is reduced after immobilizing the simulated waste resin, which helps prevent the generation of temperature cracks and enhances the durability of the solidified body. This guarantees that the MSHC-solidified body demonstrates outstanding compressive strength (over 7 MPa at 28 days), freeze-thaw resistance (compressive strength loss rate not exceeding 25%), soaking resistance (compressive strength loss rate not exceeding 25%), and impact resistance (no significant fractures during high-altitude falls) [32]. The results indicate that the MSHC-solidified body containing waste resins exhibits resilience against impacts during transportation and while being embedded in the environment. It demonstrates the capability to withstand temperature changes throughout the year without developing cracks, effectively preventing the release of radioactive nuclide ions into the environment.

#### 4.1.2. Influence of Incorporating Waste Resin on the Microstructural Mechanism of the MSHC-Solidified Body

Mg(OH)_2_ and M-S-H gel are the primary hydration products of MSHC. The M-S-H gel can interconnect the sheet-like, smaller-sized Mg(OH)_2_, contributing to the overall strength of the material. However, after adding waste resin, the reaction of the solidified body is delayed, which could be attributed to the boric acid on the surface of the waste resin particles. The inclusion of boric acid in the cement hinders the formation of hydration products, resulting in delayed hydration. At the same age, larger-sized Mg(OH)_2_ is the main hydration product of MSHC containing wastes resins, and the production of M-S-H gel is comparatively lower compared to MSHC [37]. This leads to the strength source of the solidified body relying solely on the overlapping between sheets, without proper cementation, and subsequently increases the porosity. Additionally, the increase in porosity was also due to the gap between the resin and the cement after dehydration shrinkage during cement hydration. As a result, the strength of the solidified body is reduced. It is evident that the addition of waste resins merely retards the hydration process and does not destroy the hydration products of the MSHC [38,39,40]. However, more sufficient hydration leads to higher amount of hydration products, greater strength of the solidified body, and stronger containment of solidified nuclide ions [41]. If there is a higher demand for strength, the hydration reaction can be accelerated through the use of additives.

### 4.2. Factors Affecting Ion Leaching Performance and Leaching Models on MSHC-Solidified Body Containing Waste Resins

#### 4.2.1. Factors Affecting Ion Leaching Performance on MSHC-Solidified Body Containing Waste Resins

Many studies show that MSHC exhibits superior leaching resistance compared to OPC [26,30]. This achievement effectively prevents the diffusion of internal nuclide ions into the atmosphere during and after the immobilization process of waste resins, thereby preventing environmental pollution and upholding the purpose of immobilization [42].

The leaching temperature significantly impacts the initial process of ion leaching. During this stage, ions primarily leaches out through the pore solution. Higher temperatures accelerate the migration rate of ions in the solution to the external environment, leading to an increase in the leaching rate. In the later stage of leaching, the influence of temperature on the leaching rate diminishes. This is due to the solidified body develops resistance to ion leaching, resulting in a reduced impact of temperature on the process.

The leaching rate of Cs^+^ and Sr^2+^ in deionized water is lower compared to when the leaching agent is simulated seawater, indicating that the leaching solution significantly influences the ion leaching process. The presence of chloride salts in the simulated seawater can corrode the inner matrix of the cement through the pore solution, resulting in an increase in porosity and enhancing the potential for ion migration, thereby increasing the leaching rate.

Combining the two leaching conditions, it is evident that the impact of the leaching solution on the ion leaching rate is more pronounced in comparison to the leaching temperature. The leaching rate of Cs^+^ exceeds that of Sr^2+^ marginally, owing to variations in their ability to be adsorbed and immobilized, attributes to the different radii of Cs^+^ and Sr^2+^ ions. The addition of waste resins results in a decrease in heat release, which reduces the outward migration rate of ions in nuclear waste. This observation implies that the use of MSHC helped to mitigate the potential environmental impact of nuclear waste by minimizing the release of radioactive ions into the surrounding ecosystem.

#### 4.2.2. Leaching Models of MSHC-Solidified Body Containing Waste Resins

Different isotopes possess distinct leaching models, with composite model equations being more adept at precisely determining the leaching mechanisms of Cs^+^ and Sr^2+^ [6]. As a result, this study employed fitting of the relevant composite models [43,44,45] to calculate the leaching parameters of Cs^+^ and Sr^2+^ in the MSHC-solidified body.

First-order reaction model (FRM):

(7) P_t=Q_0 (1−exp(−kt))+C

where *P_t_* represents the cumulative leaching fraction of Cs^+^ and Sr^2+^ (cm), *Q*_0_ stands the initial amount of Cs^+^ and Sr^2+^ (mg/g), *k* denotes the rate constant (d^−1^), and *C* signifies the loosely bound state nuclides (cm). The FRM depicts the ongoing ion diffusion between the solidified body surface and the leaching agent. The rate of ion exchange is primarily determined by the first-order reaction rate and the freely available ions on the solidified body surface.

First-order reaction/diffusion model (FRDM):

(8) $$P_{t}=Q_{0}\left(1-exp(-kt)\right)+2\left(\frac{S}{V}\right)\sqrt{\frac{Dt}{\pi }}+C$$

where *S* represents the surface area of the solidified body (cm^2^), *V* stands the volume of the solidified body (cm^3^), and *D* denotes the diffusion coefficient (cm^2^/d). The FRDM illustrates leaching of nuclides caused by both ion diffusion between the solidified body surface and the leaching agent and ion diffusion within the cementitious matrix itself.

Dissolution/Diffusion Model (DDIM):

(9) $$P_{t}=\left(\frac{S}{V}\right)\left(2\sqrt{\frac{Dt}{\pi }}+U_{0}\times t\right)+C$$

where *U*_0_ represents the maximum network dissolution rate (cm/d). The DDIM portrays the leaching of nuclides as being governed by both dissolution and diffusion controlled by Fick’s second law.

First-order reaction/dissolution model (FRDIM):

(10) $$P_{t}=Q_{0}\left(1-\exp\left(-kt\right)\right)+\left(\frac{S}{V}\right)\left(U_{0}\times t\right)+C$$

The FRDIM reflects the leaching of nuclides caused by ion diffusion between the solidified body surface and the leaching solution, as well as dissolution controlled by the network dissolution rate.

First-order reaction diffusion dissolution model (FRDDIM):

(11) $$P_{t}=Q_{0}\left(1-\exp\left(-kt\right)\right)+\left(\frac{S}{V}\right)\left(2\sqrt{\frac{Dt}{\pi }}+U_{0}\times t\right)+C$$

The FRDDIM results in the leaching of nuclide ions through the superposition of the FRM and the DDIM.

**Figure 12:**
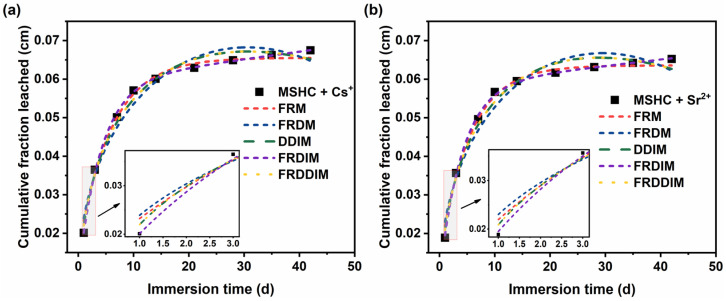
Fitting curves of different leaching models for the MSHC-solidified body containing waste resins: (**a**) MSHC + Cs^+^, and (**b**) MSHC + Sr^2+^.

The experimental results of cumulative leaching fractions of Cs^+^ and Sr^2+^ from MSHC-solidified body containing waste resins, under leaching conditions with deionized water at 25 °C, at different leaching times were obtained. The fitting was performed using the aforementioned models, and the fitted curves were presented in Figure 12. The model fitting results and parameters were detailed in Table 6.

From the fitting results, it is evident that the leaching of Cs^+^ and Sr^2+^ from the MSHC-solidified body containing waste resins aligns well with the five models. However, for the FRDM, DDIM, and FRDDIM sets of models, the fitted data yield negative values for *k*, *C*, or *U*_0_, which contradicts reality. Consequently, these models are unsuitable for simulating Cs^+^ and Sr^2+^ leaching from the MSHC-solidified body. On the other hand, the FRDIM exhibits higher correlation coefficients (*R*^2^) after fitting for various systems and different nuclide ion leaching, namely 0.99871, 0.99859, 0.99658, and 0.99860. Compared to the FRM, these coefficients are closer to 1. Therefore, the FRDIM is more appropriate for describing the leaching process of Cs^+^ and Sr^2+^ from the MSHC-solidified body. This implies that the leaching of Cs^+^ and Sr^2+^ from the MSHC-solidified body is primarily governed by ion diffusion between the solidified body surface and the leaching solution, as well as dissolution controlled by the network dissolution rate.

Furthermore, by comparing the parameters obtained from fitting the FRDIM, it can be observed that the amount of loosely bound state nuclides in the MSHC-solidified body containing Cs^+^ waste resins is 0.00873 cm, whereas for the MSHC-solidified body containing Sr^2+^ waste resins, it is 0.00795 cm. This represents an increase of 9.81% in Cs^+^ compared to Sr^2+^, indicating that Cs^+^ exhibits a higher migration and leaching capacity than Sr^2+^.

### 4.3. Immobilization Mechanism of MSHC on Waste Resins and Nuclide Ions

The leaching rate, mechanical properties, and durability after immobilization are critical indicators to ensure an effective sealing effect. The leaching resistance of the nuclide ions adsorbed on the resin can be influenced by the composition of the matrix: when the MSHC releases heat during hydration, the cumulative heat flow is low and the heat flow rate remains stable, which helps reduce the rate of nuclide migration from the pore solution to the surface of the solidified body. The presence of more gel pores in the MSHC is beneficial for the adsorption of nuclide ions, as evident from the SEM images, resulting in a reduction in the leaching rate. Simultaneously, the larger specific surface area of the hydration product of the MSHC provides a significant advantage for the adsorption of nuclide ions, and the conclusion is consistent with the experimental results of Brew [46,47]. These findings contribute to enhancing the effectiveness of immobilization and ensure improved leaching resistance for radioactive substances.

There are three main mechanisms for the immobilization of radioactive waste using a cement matrix: mechanical encapsulation, surface adsorption, and reaction with substances in the matrix to form stable insoluble compounds. These mechanisms have been mentioned in many studies [48,49,50]. Resin is a granular organic matter that does not react with the cement matrix, its immobilization in the MSHC primarily occurred through mechanical encapsulation. As for the nuclide ions in the waste resin, the absence of new phases is not revealed by XRD, SEM, and other testing methods, indicating that there is no reaction with the substances in the cement matrix. Therefore, the primary mechanisms involved in the immobilization process are mechanical encapsulation and surface adsorption. The results indicate that using MSHC to immobilize waste resins appears to be an effective method for enhancing the safety and environmental sustainability of managing nuclear waste.

### 4.4. Limitations and Applicability

While this study demonstrates the promising potential of MSHC for immobilizing waste resins, several limitations should be acknowledged:

(1) Resin Swelling Effects: The potential swelling of ion exchange resins upon water absorption may induce microcracks in the cement matrix over extended periods. Future studies should evaluate long-term dimensional stability.

(2) Chloride-Rich Environments: Although seawater leaching tests were conducted, the long-term behavior in high-chloride environments typical of geological repositories requires further investigation.

(3) Radiation Effects: This study used simulated waste resins; actual radioactive resins may undergo radiolytic decomposition, potentially affecting the immobilization performance.

For real-world implementation, pre-saturation of resins to minimize swelling and the use of supplementary additives to enhance chemical resistance are recommended.

## 5. Conclusions

In this study, the efficacy of MSHC in immobilizing simulated waste resins was evaluated, and the impact of waste resins on the hydration process and the composition of hydration products of MSHC was investigated. The key findings of this study can be summarized as follows:

(1) MSHC-solidified body containing waste resins exhibits satisfactory compressive strength, impact resistance, freeze-thaw resistance, and soaking resistance performance. These results indicate that MSHC holds great potential for the future practical applications in immobilizing waste resins containing nuclide ions.

(2) The incorporation of waste resins leads to a delayed onset of the exothermic peak, a postponement of the hydration reaction, and an increase in porosity. This incorporation does not disrupt the hydration products; rather, it leads to a reduction.

(3) Different leaching conditions will affect the leaching process of Cs^+^ and Sr^2+^, and the type of leaching solution has a more pronounced impact on the leaching rate of nuclide ions. The leaching rate of Cs^+^ is slightly higher than that of Sr^2+^ due to their different radii.

(4) The leaching of Cs^+^ and Sr^2+^ from MSHC-solidified body follows the FRDIM. This suggests that the leaching of these two nuclide ions is primarily controlled by ion diffusion at the interface between the solidified body and the leaching solution, as well as dissolution controlled by the network dissolution rate.

(5) In the case of waste resins, MSHC employs mechanical encapsulation. For the nuclide ions within the waste resins, MSHC utilizes both mechanical encapsulation and surface adsorption mechanisms.

## Figures and Tables

**Table 1:** Chemical composition of raw materials.

Composition (wt.%)	MgO	CaO	SO_3_	SiO_2_	P_2_O_5_	Fe_2_O_3_	Al_2_O_3_	MnO	K_2_O	Na_2_O
MgO	95.80	2.04	0.73	0.26	0.15	0.12	0.09	0.02	0.01	—
SF	0.26	0.83	0.15	97.09	0.24	0.11	0.35	0.03	0.80	0.12

**Table 2:** The mass and compressive strength loss rate of solidified body before and after freeze-thaw test.

Category	Mass Loss Rate (%)	Compressive Strength Loss Rate (%)
MSHC	0.12	9.41
MSHC + Cs^+^	0.26	12.69
MSHC + Sr^2+^	0.24	9.83
MSHC + Cs^+^/Sr^2+^	0.32	11.85

**Table 3:** The compressive strength loss rate of the solidified body before and after the soaking test.

Category	Before Soaking (MPa)	After Soaking (MPa)	After Soaking/Before Soaking
MSHC	45.36	54.22	1.20
MSHC + Cs^+^	14.42	15.63	1.08
MSHC + Sr^2+^	13.15	14.60	1.11
MSHC + Cs^+^/Sr^2+^	9.48	11.83	1.25

**Table 4:** Content of Mg(OH)_2_ and M-S-H gel in the solidified body at different ages.

Curing Age (Days)	Category	Content (%)	
		Mg(OH)_2_	M-S-H Gel
7	MSHC	17.953	13.790
	MSHC + Cs^+^	21.413	13.412
	MSHC + Sr^2+^	22.742	11.153
	MSHC + Cs^+^/Sr^2+^	18.459	13.128
28	MSHC	12.231	21.283
	MSHC + Cs^+^	13.924	20.051
	MSHC + Sr^2+^	17.546	19.878
	MSHC + Cs^+^/Sr^2+^	15.429	21.132

**Table 5:** Analysis of pore structure of the solidified body.

Category	Average Pore Size (nm)	Median Pore Size (nm)	Total Pore Area (m^2^/g)	Porosity (%)
MSHC	21.69	7.20	31.97	15.28
MSHC + Cs^+^	34.40	19.80	62.68	37.87
MSHC + Sr^2+^	58.30	19.10	40.09	42.11
MSHC + Cs^+^/Sr^2+^	27.60	14.50	71.95	35.14

**Table 6:** Fitting results and parameters of different leaching models.

Category	Leaching Models	Parameters				*R* ^2^
		*k*	*C*	*D*	*U*_0_	
MSHC + Cs^+^	FRM	0.17825	0.02139	-	-	0.99124
	FRDM	−0.01901	0.01154	1.68759 × 10^−4^	-	0.96855
	DDIM	-	−1.511 × 10^−4^	3.19733 × 10^−4^	−0.00247	0.98675
	FRDIM	0.23982	0.00873	-	1.72368 × 10^−4^	0.99883
	FRDDIM	−1.8459 × 10^−6^	−1.51279 × 10^−4^	3.19735 × 10^−4^	−0.00247	0.98675
MSHC + Sr^2+^	FRM	0.18426	0.01298	-	-	0.99354
	FRDM	−0.01877	0.00663	1.91572 × 10^−4^	-	0.96887
	DDIM	-	−0.00237	3.62276 × 10^−4^	−0.00289	0.98425
	FRDIM	0.34668	0.00795	-	1.42095 × 10^−4^	0.99891
	FRDDIM	−1.348752 × 10^−5^	−0.00237	3.62287 × 10^−4^	−0.00289	0.98425

## Data Availability

The original contributions presented in this study are included in the article. Further inquiries can be directed to the corresponding authors.
